# Flapless Immediate Implants: Soft Tissue Alterations Following a Trimodal Approach with or Without Modifying Osseous and Mucosal Compartments in the Esthetic Zone: A Non-Randomized Clinical Trial with Historical Control Group

**DOI:** 10.3390/dj13100478

**Published:** 2025-10-17

**Authors:** Gustavo Cabello, María Rioboo, Daniel Torres-Lagares, Javier Fábrega

**Affiliations:** 1 Private Practice of Periodontics, Prosthodontics, and Implants, 29008 Málaga, Spain; info@clinicanexus.com; 2 Department of Clinical Specialties, School of Dentistry, Complutense University of Madrid (UCM), 28040 Madrid, Spain; 3 Department of Oral Surgery, Dental School, University of Seville, 41009 Seville, Spain; 4 Private Practice of Prosthodontics, 28010 Madrid, Spain; jfabrega@drfabrega.com

**Keywords:** dental implants, immediate implant placement, soft tissue graft, connective tissue, tissue remodeling, provisional implant restoration, dental prosthesis, implant-supported, trimodal approach, combined modality therapy

## Abstract

**Objective**: This study aimed to compare two protocols for immediate implants with fixed provisional restoration, no grafting (trimodal approach = TA) versus grafting in both the osseous gap and peri-implant mucosa (a trimodal approach with modification of the bony and mucosal compartments = TAOM), by measuring soft tissue changes over time. The periodontal phenotype was noted to investigate the relationship between its thickness and the clinical outcomes. **Methods**: Thirty-one patients met the inclusion criteria (15 in the TA group and 16 in the TAOM group). The TA group was a historical control group. Measurements were taken using a digital caliper at T0 and 3, 6, and 12 months following the procedure (T3), (T6), and (T12), respectively, from reference points marked in a dental-supported stent. The periodontal phenotype was determined using an analogical caliper. **Results**: T12: Vertical midfacial change was −0.17 ± 0.37 in the TAOM group and 0.54 ± 0.33 in the TA group, respectively. Statistical significance (*p* = 0.0001) was found. Papilla vertical change in the TAOM group was −0.16 ± 0.45 mesially and 0.00 ± 0.44 distally. In the TA group, it was 0.55 ± 0.82 mesially and 0.86 ± 0.95 distally. Statistical significance (*p* = 0.0001) was also found. **Conclusions**: There were differences in soft tissue change between the two groups, and changes were related to the periodontal phenotype. Studies with more extended follow-up periods are needed to assess the long-term evolution of both protocols.

## 1. Introduction

**Figure 1:**
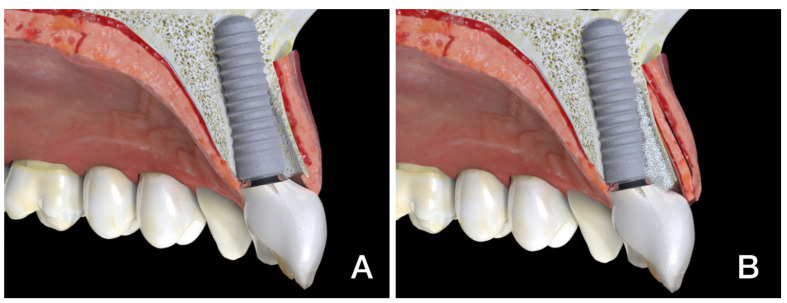
(**A**) TA: Trimodal approach with no graft. (**B**) TAOM: Trimodal approach with osseous and mucosal graft.

Flapless immediate implant placement with immediate provisionalization (trimodal approach, TA) (Figure 1A) is now considered a positive approach to specific clinical situations due to its less invasive and simpler clinical protocol, together with high rates of acceptance by patients according to comfort, length of treatment, and esthetic outcome [1,2,3,4,5]. Many studies have shown this trimodal protocol to be reliable and predictable in selected clinical circumstances, leading to widespread indication for and acceptance of this technique, which is considered a reasonable option for clinicians and patients, since short- and mid-term esthetic results compare favorably with those of delayed protocols in the proposed clinical scenarios [1,2,3,4,5,6,7,8,9,10].

However, questions have arisen regarding the recession of midfacial mucosa and papillae when implementing this approach. In this sense, compensatory and preventive actions in the bone (gap filling) and mucosal compartments (addition of connective tissue grafts) have been proposed. This latter procedure could be called the “mucosal and osseous trimodal approach” (TAOM) as it includes immediate flapless post-extraction implantation and provisional restoration placement with osseous and mucosal modification in one single clinical procedure (Figure 1B).

Although the authors of this study and several other research groups have recently published acceptable clinical results achieved with the trimodal approach (TA) [1,2,3,4,5,6,7,8,9,10], the patient follow-up time has been limited (usually 12 months). Furthermore, the question of whether better and/or more stable outcomes can be achieved through the implementation of additional interventions in the osseous compartment (TAO) [6,7,8,11] and the mucosal compartment (TAOM) [9,12,13,14] warrants further investigation.

In relation to the grafting of the osseous compartment (TAO), a very recent systematic review [6] found that socket grafting is accepted as the standard of care in immediate implant placement, as it is a simple procedure and has been shown to preserve bone volume [6,7,8].

Regarding surgical intervention in the mucosal compartment (TAOM), a recent systematic review [9] provided, based on the GRADE guidelines, a moderate recommendation for the use of a connective tissue graft (CTG) following immediate implant placement. Although there seems to be a trend of grafting the mucosa around immediate implants and restorations, with many clinical studies in this field available, many questions remain. Does TAOM produce better and longer-term biological and esthetic outcomes when compared to TA or TAO? Should TAOM be routinely implemented, and what would be the patient selection criteria? Would it be an option to graft the soft tissue if the final restoration has been in place for several months and the esthetic outcome is not acceptable? It should be noted that soft tissue grafting, as opposed to grafting of the osseous gap in the alveolar socket, is a technically demanding procedure in terms of both treatment time and morbidity. The financial implications of this intervention must also be taken into account.

At this point, there are a limited number of controlled clinical studies addressing these questions, particularly when it comes to comparing the results between flapless immediate implants, with or without changing both the hard and soft tissue compartments, and data comparing the effect of different protocols on the midfacial mucosa and papillae height around the provisional and final restorations of such implants.

The aim of this clinical study is twofold: First, we aim to determine the effect of a protocol, in terms of both vertical midfacial and papillae linear soft tissue changes, in which both bone compartments (alveolar gap filling) and mucosal compartments (connective tissue graft) are grafted immediately following flapless implant placement (TAOM) to a control group in which no gap filling or connective tissue graft is implemented (TA). Second, we aim to identify any correlation between these changes and the patient’s periodontal biotype (phenotype).

## 2. Materials and Methods

### 2.1. Study Design

This is a clinical trial comparing two consecutive case series, i.e., with two non-parallel groups. We used the group from our first study, published in January 2013, as a control group, comparing it with a new series of patients. The study protocol for this new series of patients was approved by the CEIm Quiron Salud Catalunya Institutional Research Ethics Committee (Approval code: 2022/102_MAX: CEX, on 2 March 2023). The study was registered at ClinicalTrial.gov (NCT05927181) and followed the CARE (Case Report) guidelines checklist. Therefore, this study includes both a historical group of 15 patients and a new group of 16 patients who were enrolled and treated at the same two private practices (GC and JGF). All eligible patients were consecutively selected and received immediately placed flapless implants with provisional restoration from 2006 to 2012 (for the historical control group) and from 2013 to 2018 (for the new series of cases).

### 2.2. Patient Population

Patients in need of single-implant-supported restorations in the anterior maxilla (the canines, lateral, and central incisors) were selected for this study. The criteria for tooth extraction were extensive non-restorable caries, endodontic complications (e.g., root fracture), internal resorption, and restorative (ferrule) limitations. Patients in both the historical control group and the new case series had to fulfil the inclusion and exclusion criteria described below.

Inclusion criteria:

- Age ≥ 18 years;
- Requiring tooth extraction in the anterior maxilla (13–23) due to the reasons described above;
- A failing tooth with adjacent and opposing natural teeth;
- Adequate oral hygiene (bleeding on probing < 20%; plaque index < 20%);
- The absence of active and uncontrolled periodontal disease;
- Sufficient mesial–distal and interocclusal space for implant placement and definitive restoration;
- Sufficient interocclusal space to design a non-occluding provisional restoration;
- Integrity of all bone walls around the problem tooth.
- Exclusion criteria:
- Systemic metabolic or osseous disease that could compromise peri-implant tissue healing;
- Acute infection in the treatment area.
- The absence of one or both adjacent teeth.

Lack of integrity of the buccal bone wall (dehiscence or fenestration) was observed during surgery or when tooth extraction altered the integrity of the osseous and gingival architecture.

The participants were screened by two investigators (GC and JGF) and comprised two consecutive study groups with the following interventions: (1) flapless immediate implant placement and provisional restoration (trimodal approach: TA) (*n* = 15) 13; (2) flapless immediate implant placement and provisional restoration with alveolar filling and a connective tissue graft (a trimodal approach with osseous and mucosal compartment modification: TAOM) (*n* = 16).

### 2.3. Treatment Protocol and Follow-Up

**Figure 2:**
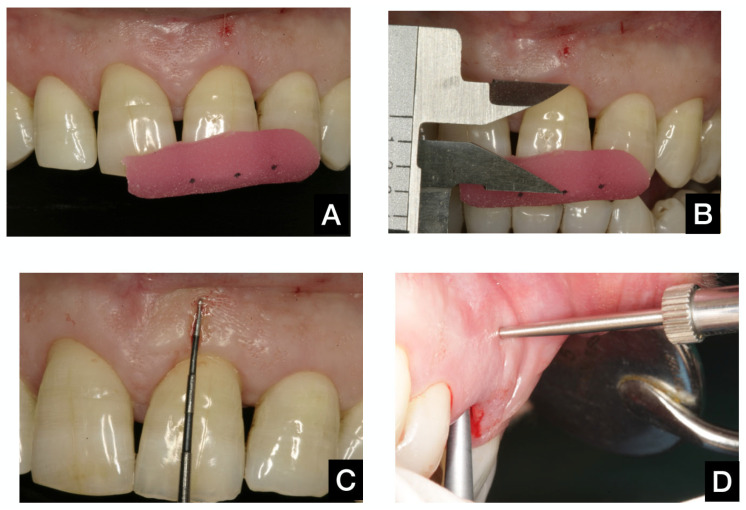
(**A**): Measuring the stent in position. (**B**): Measuring with the digital caliper. (**C**): Mucosal reference point to measure periodontium width. (**D**): Measuring periodontium width from inside the extraction socket.

Four visits were planned for each participant. Following the selection of candidates, study casts, preoperative photographs, and orthogonal periapical radiographs of the tooth problem were prepared for all patients. Rigid stents were prepared with light-cured resin, covering the incisal edges of at least one adjacent tooth on each side of the target tooth. Measurements from reference points to mid-buccal soft tissue height and mesial and distal papillae were recorded at T0. The stents were more than 2 mm thick, allowing them to be modified for a perfect fit over the final restoration, as shown in Figure 2A,B.

All surgical procedures were performed by two experienced surgeons and investigators (GC and JGF). Without raising a mucoperiosteal flap, careful tooth extraction was performed, allowing for the preservation of soft and hard tissues. In 10 patients, the Benex Extractor^®^ (Benex Root Extraction System, Hager and Meisinger GmbH, Neuss, Germany) was used to ensure atraumatic extraction. Then, a detailed examination of the buccal bone wall was carried out to verify its integrity. Patients in whom the integrity of the bone wall was questioned were excluded from the study, and the implant was placed using conventional protocols. The total periodontal thickness (phenotype) was measured at this time using an analog caliper located 5 mm apical to the buccal gingival margin to find the bundle bone level (Figure 2C,D).

Next, the implant bed was prepared on the palatal side of the extraction socket in accordance with the manufacturer’s instructions (Bone Level -BL- and Tissue Level -TL-, Straumann AG, Basel, Switzerland). The implant was placed to secure the ideal position at a depth of 2 mm for the TL implant and 4 mm for the BL implant, in a palatal position, allowing for direct screw-retained restoration (1–3 mm from the bone wall) for all implants. It was also verified that the wall of the post-extraction socket was not perforated during implant bed preparation. The implant had to show good primary stability in its ideal position to avoid being excluded from the study. In the TA group, no material was used to fill the gap between the implant and the osseous wall, and no soft tissue graft was attempted. In the TAOM group, the gap between the implant and the facial bone wall was grafted with inorganic bovine bone (Geistlich Bio-Oss^®^; Geistlich Pharma AG, Wolhusen, Switzerland); in addition, a connective tissue graft (CTG) was harvested from the anterior palate region and placed submucosally on the labial bone plate using the envelope technique. The recipient submucosal site for the CTG in the TAOM group was prepared at full thickness using a micro-periostotome with sufficient overextension to allow for tension-free graft placement.

Following implant placement, an acrylic resin implant-supported provisional restoration was prepared, adapted, and implemented for all implants. All provisional restorations were directly screw-retained and designed to have no occlusal contact in the maximum intercuspal position or during excursive movement. The emergence profile was mainly flat or concave on the interproximal and palatal sides and slightly convex (anatomical emergence profile) in the buccal aspect to support the TA group’s soft tissues. In the TAOM group, the emergence profile was somewhat concave, providing space for the soft-tissue graft and avoiding excessive pressure on the marginal mucosa.

Intra-oral radiographs were taken to check the implant’s position and the fit of the restoration. Systemic antibiotics (Azitromycin-500 mg/24 h/3 days) and anti-inflammatories (Dexketoprofen-25 mg/8 h/3 days) were prescribed, in combination with clorhexidine 0.20% gel, to be used twice a day for one week. The patients were then recalled for a post-operative check-up, and sutures were removed after 14 days. The exact post-operative instructions and prescriptions were given to both groups.

At 3 months, final impressions were made. After the abutment selection and bisque try-ins, the final restorations were permanently placed four months later.

The tooth-supported acrylic resin measuring stent was then adapted to ensure a complete fit on the incisal edges of both neighboring teeth, thereby providing a reliable positioning and measurement reference.

The first measurement was taken on day 0 (immediate implant placement and provisionalization). Patient recall appointments for examinations and measurements took place at 3, 6, and 12 months. No further actions were taken to increase patient compliance beyond good clinical practice protocols.

**Figure 3:**
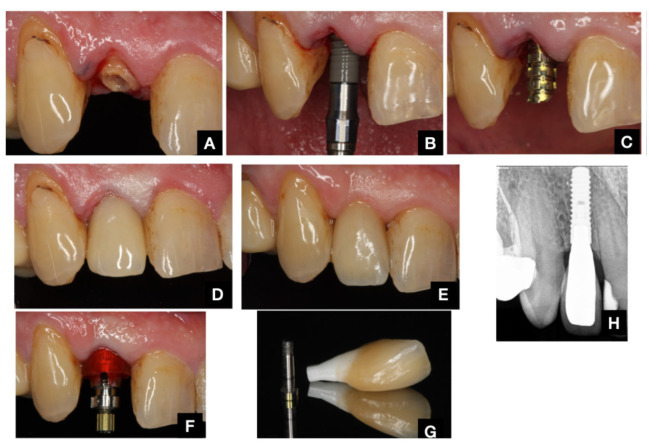
TA protocol. (**A**): Presurgical situation. (**B**): Immediate implant placement. (**C**): Provisional abutment in place. (**D**): Provisional acrylic restoration in place (T0) (**E**): Provisional restoration (T = 3). (**F**): Final impression after 3 months. (**G**): Final restoration. (**H**): Final radiograph after 12 months.

**Figure 4:**
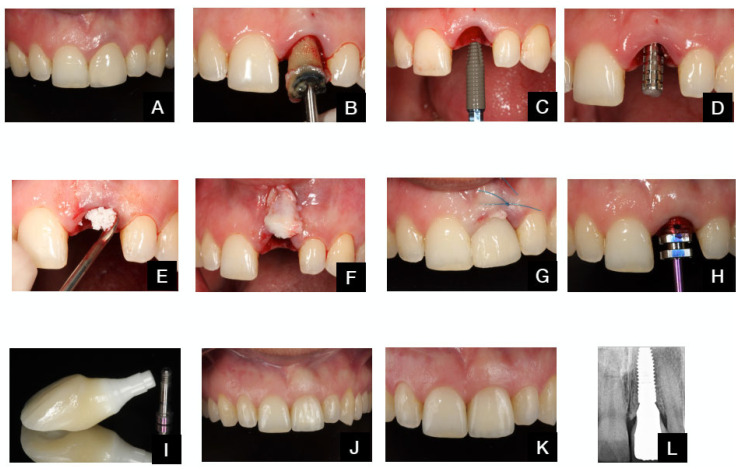
TAOM protocol. (**A**). Pre-op situation. (**B**). Tooth extraction. (**C**). Immediate implant placement. (**D**). Provisional abutment. (**E**). Bone graft in the del buccal gap. (**F**). Connective tissue-free palatal graft before insertion in tunnel preparation. (**G**). Grafts and provisional restoration in place. (**H**). Final impression. (**I**). Final restoration. (**J**). Final restoration delivered after 4 months. (**K**). Outcome after 12 months. (**L**). Final radiograph after 12 months.

The various clinical protocols are illustrated in Figure 3 and Figure 4.

### 2.4. Outcome Measures

The primary outcome variable of this study was the vertical midfacial soft tissue change from baseline (before tooth extraction) to 12 months following implant placement. The secondary outcome measures were vertical changes in mesial and distal papillae, the periodontal phenotype at the implant site, and mechanical complications.

The clinical outcomes were measured as follows:

Vertical soft tissue changes were calculated by measuring the distance from the reference measuring points on the stent to mesial (MP) and distal (DP) papillae and the gingival zenith (Z) of the implant restoration. Distances were measured using a precision digital caliper (Mitutoyo, Kanagawa, Japan) that can display measurements as small as tenths of a millimeter. Three dimples were marked in the stent, along the approximate vertical projection of the papillae and zenith (Figure 2A,B). Measurements were taken before tooth extraction (baseline) at T0 and at 3 (T3), 6 (T6), and 12 (T12) months following the intervention.

Periodontal phenotype thickness was measured using an analogical caliper (Mitutoyo, Kanagawa, Japan) able to measure accurately to tenths of a millimeter. It was located inside the post-extraction socket, 5 mm apical to the buccal gingival margin, immediately following tooth extraction (Figure 2C,D).

The following mechanical and biological complications were assessed and included: screw loosening, loss of retention, chipping of acrylic or porcelain, implant failure, and infections.

### 2.5. Statistical Analysis

The unit of study for the analysis was the patient. For all the variables, a descriptive study was carried out based on means, standard deviations, and 95% CIs for quantitative variables (the previous determination of sample distribution) and values of prevalence and CI 95% for the categorical variables.

ANOVA was applied for numerical variables with a normal distribution. For the variables that did not demonstrate a normal distribution, the Mann–Whitney U test was used. Categorical variables were analyzed using the Chi-square test. For variables with non-normal distribution, the Pearson or Spearman correlation was applied to determine relationships between numerical variables.

All analyses were conducted using SPSS (version 23.0; SPSS Inc.; IBM Corporation, Chicago, IL, USA), with a *p*-value of 0.05 used to determine statistical significance.

## 3. Results

### 3.1. Sample Description

**Figure 5:**
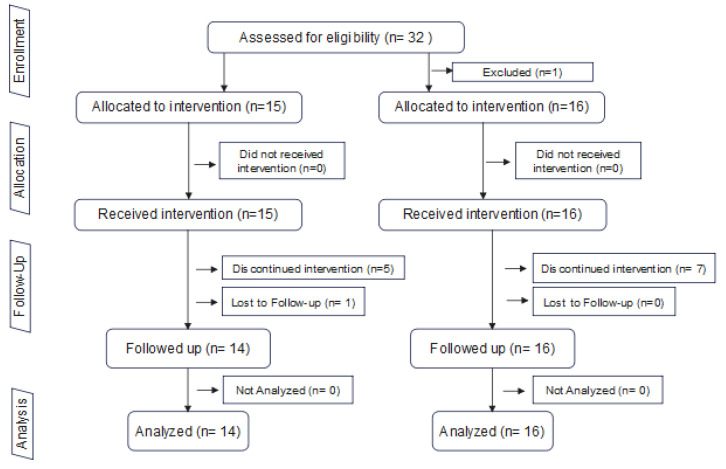
Study flowchart.

Thirty-one patients participated in the study: fifteen were enrolled in the TA group and sixteen were enrolled in the TAOM group. There was one dropout in the TA group (the patient moved out of the country before the final recall appointment). The study flowchart is shown in Figure 5.

Details regarding patient characteristics at baseline are shown in Table 1. Teeth were extracted because of root fractures (38.7%), crown fractures (25.8%), internal root resorption (9.7%), endodontic failure (9.7%), the absence of ferrule (9.7%), or radicular decay (6.5%).

All patients tolerated the surgical procedures well, fully complying with the study protocol and experiencing no postoperative complications. No biological complications were noted in either group. In the TA group, ten of the crowns were metal-ceramic and directly screw-retained; three were zirconia-based crowns cemented on a prefabricated Zr abutment (Straumann Anatomical IPS e. max Abutment [Institute Straumann AG, Basel, Switzerland]); and two customized Zr anatomic abutments layered with esthetic ceramic and directly screw-retained (CARES System, Institute Straumann AG).

In the TAOM group, all crowns were screwed directly onto the implant, including five crowns on custom-made zirconia abutments (CARES System, Institute Straumann AG, Basel, Switzerland) and 11 zirconia–ceramic crowns cemented on VariobaseTM abutments [Institute Straumann AG]. One implant in the TAOM group experienced a mechanical complication (screw loosening).

#### 3.1.1. Vertical Soft Tissue Changes

The mean (±SD) changes in the measurements when evaluating the vertical midfacial soft tissue position between T0 and T12 were −0.17 ± 0.37 in the TAOM group and 0.54 ± 0.33 in the TA group, respectively. The mean (±SD) difference between groups at T12 was 0.71 mm, and statistical significance (*p* < 0.0001) was found.

The mean (±SD) changes for the evaluation of mesial and distal vertical papillae height between T0 and T12 were −0.16 ± 0.45 and 0.00 ± 0.44 for the TAOM group and 0.55 ± 0.82 and 0.86 ± 0.95 for the TA group, respectively. The mean (±SD) differences between groups at T12 were 0.71 mm for mesial papillae and 0.86 mm for distal papillae; statistical significance was found for both groups.

The resulting changes in soft tissue position at T3, T6, and T12 are shown in Table 2. Comparisons are made between the groups in Table 3.

#### 3.1.2. Periodontal Phenotype Thickness

When both groups were assessed together, the mean (±SD) for the thin periodontal phenotype (≤1.5 mm) was 1.21 ± 0.19, and the mean for the thick phenotype (>1.5 mm) was 1.93 ± 0.33.

The mean (±SD) thicknesses of the periodontal phenotypes at T0 were 1.4 ± 0.33 and 1.68 ± 0.51 for the TAOM and TA groups, respectively.

Significant differences (*p* < 0.05) were found at T3 in the TAOM group for facial soft tissue changes between the thin (−0.36 ± 0.32) and thick (0.03 ± 0.34) phenotypes. However, no statistically significant differences were observed at any time point between the two phenotypes in the TA group.

#### 3.1.3. Correlation Between Vertical Soft Tissue Changes with Phenotype and Gingival Width

A positive correlation was found between vertical soft tissue changes and phenotype, as well as gingival width, at T12 when both groups were analyzed together. The association was found to be statistically significant for phenotype.

This correlation was negative in the TA group at T12, i.e., the greater the soft tissue width and thickness at baseline, the fewer vertical soft tissue changes were observed. However, this association was not statistically significant.

In the TAOM group, this correlation was positive at T12. The greater the soft tissue width and thickness at baseline, the more vertical soft tissue alterations were observed (in terms of overgrowth). Once more, this association was not statistically significant. Data regarding the correlations are shown in Table 4.

**Figure 6:**
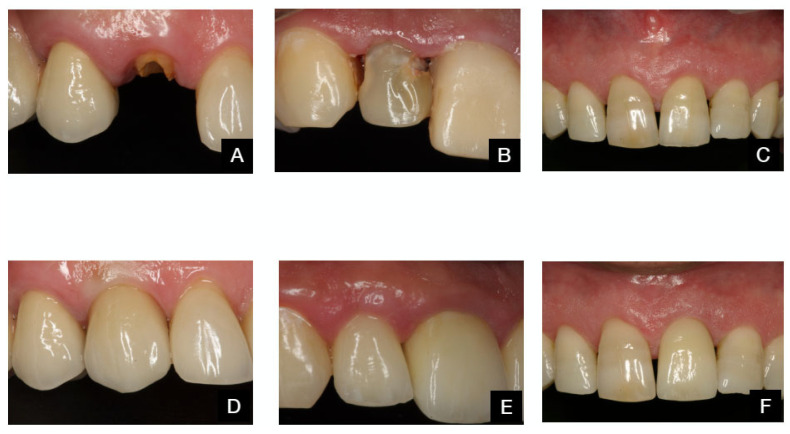
Before and after TA protocol. Patient 1: (**A**,**D**). Patient 2: (**B**,**E**). Patient 3: (**C**,**F**).

**Figure 7:**
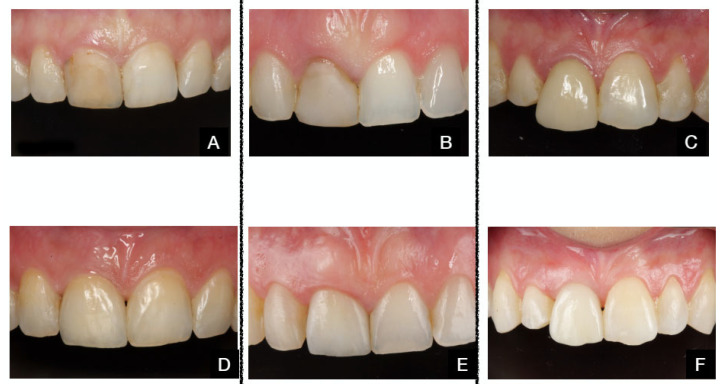
Before and after TAOM protocol. Patient 1: (**A**,**D**): Patient 2: (**B**,**E**). Patient 3: (**C**,**F**).

The values before and 12 months after the TA and TAOM protocols are shown in Figure 6 and Figure 7.

## 4. Discussion

This non-randomized clinical study compared two treatment options: immediate post-extraction implant placement with immediate fixed provisional restoration and no grafting of the osseous gap or peri-implant mucosa (TA) versus immediate post-extraction implant placement with immediate fixed provisional restoration and grafting of both the osseous gap and peri-implant mucosa (TAOM). Statistically significant differences were found in soft tissue changes and their relationship to the patient’s phenotype. Our results are consistent with those of previous studies, which suggest that connective tissue grafting at the time of immediate implant placement may minimize or compensate for mid-facial soft tissue apical migration [12,13,14].

Our findings also agree with authors who have reported significant differences in clinical outcomes when initial periodontal phenotypes are compared [9,12].

Although an increasing body of clinical research has been conducted on this topic, several limitations regarding the design of the studies and the availability of long-term data to support this clinical protocol should prevent clinicians from considering a soft tissue graft the standard of care when placing immediate implants. In this sense, the most recent and comprehensive meta-analysis available only moderately recommends TAOM procedures due to the issues experienced when comparing data from the available systematic reviews [9].

Digital tools were not readily available when the first patient group was treated. These tools are now widely used and are especially valuable for assessing volumetric changes. As we only measured the linear distance between two points in a very accessible area with an exact tool, the measurements were deemed precise and consistent. The second group required the same protocol used in the first group to enable a valid comparison.

In the most recent consensus report published by the DGI/SEPA/Osteology Workshop (aimed at elucidating key aspects related to peri-implant soft tissues and their role in maintaining health and esthetic outcomes in implant dentistry), various measurement techniques employed in the included clinical studies were described. These ranged from linear measurements (in millimeters) taken with periodontal probe, endodontic file, ultrasonic devices, and cast models, with only one study utilizing CBCT imaging [15]. Regarding the use of analogical recording methods, the current consensus reflects that no established guide or standard states that digital methods are more accurate than analogical ones, particularly in linear measurements, as is the case in our study.

Clinicians are continually faced with the task of treating their patients in the most efficient and safe manner, following the protocol that yields the most stable clinical outcome, based on the available body of scientific evidence. Thus, the clear trend observed in the reviewed literature regarding the benefits of soft tissue grafting when implemented together with immediate implants in the esthetic zone should not be overlooked.

The next step is to identify clinical scenarios where a connective tissue graft (CTG) would be indicated. The results of our study, consistent with many others [9,12,13,14,16,17,18,19,20,21,22,23,24,25,26], highlight the initial periodontal phenotype of the patient as a relevant factor. Since the periodontal phenotype classification based on thickness is ill-defined and the intervals are unquantified, we divided our groups into thin (≤1.5 mm) and thick (>1.5 mm) phenotypes. Interestingly, both phenotype groups exhibited a sizable but comparable apical migration of midfacial soft tissues in the non-grafted group (TA) (thin: 0.51 ± 0.42 mm; thick: 0.55 ± 0.26 mm). This may be related to the observation that thin phenotypes seem to be highly affected by open-flap procedures but not by flapless interventions. Another contributing factor could be immediate provisional restoration, which hypothetically acts as a “restorative tissue inhibitor” [27] and plays a vital role in the healing and stability of the soft tissue [1,17,27]. Other factors may include the limited sample number (*n* = 14), the short follow-up time (12 months), and the presence of a thicker phenotype around the implant restorations compared to the thin periodontium present before extraction and implant procedures took place. Conversely, patients in the grafted group (TAOM) with both thin (0.30 ± 0.34) and thick (0.04 ± 0.35) phenotypes showed minimal changes in soft tissue levels. However, a positive correlation was found between vertical soft tissue changes and phenotype, as well as gingival width, at T12 when both groups were analyzed together. The association was statistically significant for phenotype (Table 4).

In light of the available data, and in agreement with Cosyn et al. [18], it appears that patients with a thinner phenotype may benefit from CTG (TAOM) at the time of implant placement, as evidenced by reduced apical migration at the mid-buccal mucosal margin. However, patients with a thicker phenotype may not have a clear-cut indication for CTG, considering that this preventive/compensatory procedure entails complexity, morbidity, and additional expenses. Although a thicker tissue around the implant-supported restoration will likely result in a more stable frame in the long term, controlled studies with a longer follow-up time are not currently available to confirm this. Changes in soft tissue over long periods of time, possibly because of physiological apical migration, have been reported [10,19,20,21,22,23].

Esthetic outcomes have been reported comparing grafted vs. non-grafted groups using a modified esthetic index [24], with no relevant difference recorded between the groups [25]. Volumetric 3D changes, measured using digital tools, have shown a limited increase in soft tissue volume, rendering a PES comparable to that of the non-grafted group (TA). Two reasons may explain this finding. Firstly, the root eminence volume parameter within the PES is a frequently weak point in the esthetic zone of implant-supported restorations [26], as it has a similar volume to that before extraction, and this apical portion of the buccal aspect is not grafted during the procedure. Secondly, alveolar facial bone wall volume loss is higher when CTG is implemented compared to non-grafted patients [14]; this is likely a side effect of the surgical manipulation of the treated area. Concerns have been raised regarding the color and/or texture of the grafted area not matching the surrounding tissues [14,25] and the excessive volume often present when a CTG is implemented in patients with thick phenotypes [26]. Thus, CTG may not always be beneficial or prosperous.

Although this study does not analyze alterations in the horizontal plane (a reduction in the vestibular tissue contour), different publications conclude that the use of immediate provisional restorations, which may prevent tissue collapse at the early stages of healing, may be a relevant factor in the preservation of the tissue’s height and volume [2,5,17,19,20,28,29,30]. The design of the provisional (implant-supported) restoration appears to be a relevant factor in the outcome. A close-to-anatomical emergence profile has been suggested as a better option [28,29], compared to more basic shapes, such as cylindrical or conical [30].

An issue may arise regarding the possible influence of the provisional restoration design on the outcome of the groups. In the TA, the restoration was meant to give support to a relatively thin, non-grafted soft tissue. This was pointless in the TAOM approach. We then utilized a more concave surface in the vestibular aspect to increase room for the soft tissue graft without “stressing” the soft tissue “tunnel” with undesirable and long-lasting ischemia. This approach, which involves leaving space for soft tissues, whether original or grafted, and using a “slim” provisional restoration design, provides sufficient buccal support while retaining the option to modify contours when the tissues mature months later.

Finally, the real impact on patients’ subjective appreciation of the esthetic outcome has not been established [31]. It has been demonstrated that laypersons and clinicians struggle to identify minor changes in gingival height and other alterations in tooth position [32,33]. The numbers included in our study (around 0.5 mm) likely fit into this category. Indeed, clinical studies comparing immediate implants with and without CTC did not show a significant difference in PES/WES scores or patient satisfaction [9,23]. Interestingly, a substantial difference in soft-tissue health between grafted and non-grafted patients was also not established [9]. In our study, PES/WES scores were not utilized. It was beyond the scope of our research, which focused solely on linear changes in soft tissue heights over time, its possible relationship to patients’ periodontal phenotype, and the modality of the treatment protocol. However, the patients included in both groups were satisfied with the overall esthetic.

## 5. Limitations

The experimental design is a limitation of this study. The use of historical controls may lead to potential confounding factors and bias related to patient selection, data variability, changes in treatment and technology, and the quality of historical records. To minimize the impact of the study design, we ensured that clear and consistent criteria were used for selecting cases and that standardized data collection procedures were employed. We documented all steps of this study in detail, allowing other researchers to reproduce the research protocol. Nevertheless, the use of a historical control group is a valuable alternative in biomedical research, especially when randomized clinical trials could pose ethical conflicts [34,35,36,37].

## 6. Future Directions

Controlled studies with longer follow-up times are needed to assess and establish the long-term evolution of both grafted (TAOM) and non-grafted (TA) patients, as well as the relationship this has with their initial phenotype.

## 7. Conclusions

The results suggest that the TAOM protocol provides improved preservation of the vertical position of the peri-implant soft tissues, compared to the non-grafted TA, with vertical changes significantly reduced at 12 months.

The findings may support the selection of the TAOM protocol as a valid option when treating patients with immediate implants in the aesthetic zone.

The inclusion of a periodontal phenotype assessment is a promising approach for treatment personalization, as initial tissue thickness may influence clinical outcomes.

Randomized clinical studies with a larger sample size, extended follow-up periods, and more detailed individualization of patient characteristics are needed not only to confirm the benefits of the TAOM protocol but also to determine the specific clinical scenarios in which this treatment option may be indicated.

## Figures and Tables

**Table 1:** Patient characteristics at baseline per study group.

Variable	TAOM Group (*n* = 16)	TA Group (*n* = 15)	Sign
Male/female ratio	8/8	9/6	<0.05
Age in years <49/≥50	11/5	5/10	
Tobacco 0/<10/≥10	13/2/1	10/2/3	
Gingival phenotype ≤1.5 mm/>1.5 mm	10/6	7/8	
Gingival width ≤6 mm/>6 mm	8/8	7/8	
Implant site location Central Incisor/Lateral Incisor/Canine	9/6/1	5/7/3	

**Table 2:** Changes in vertical soft tissues from baseline (T_pre_) to T_3_, T_6_, and final assessment (T_12_).

Variable	TA		TAOM		Sign.
	Mean	S.D.	Mean	S.D.	
T_pre_−T_3_					
Mesial	0.50	0.43	0.05	0.49	<0.05
Facial	0.29	0.45	−0.21	0.37	<0.01
Distal	0.64	0.78	0.11	0.45	<0.05
T_pre_−T_6_					
Mesial	0.23	1.32	−0.41	1.04	<0.05
Facial	0.05	1.27	−0.73	1.42	<0.01
Distal	0.63	1.31	−0.34	1.07	<0.05
T_pre_−T_12_					
Mesial	0.55	0.82	−0.16	0.45	<0.01
Facial	0.54	0.33	−0.17	0.37	<0.0001
Distal	0.86	0.95	0.00	0.44	<0.01

**Table 3:** Changes in vertical midfacial soft tissues depending on periodontal phenotype TAOM and TA groups.

	TAOM				TA			
Variable	Thin Phenotype (≤1.5 mm)		Thick Phenotype (>1.5 mm)		Thin Phenotype (≤1.5 mm)		Thick Phenotype (>1.5 mm)	
	Mean	SD	Mean	SD	Mean	SD	Mean	SD
T_pre_−T_12_ Facial	−0.30	0.34	0.04	0.35	0.51	0.42	0.55	0.26

**Table 4:** Correlations between vertical soft tissues change with phenotype and width. Corr = Correlation; Sign. = Signification; * is unnecessary.

	TAOM + TA				TAOM				TA			
Variable	Width		Phenotype		Width		Phenotype		Width		Phenotype	
	Corr *	Sign.	Corr *	Sign.	Corr *	Sign	Corr *	Sign	Corr *	Sign	Corr *	Sign
T_pre_–T_12_ Facial	0.224		0.355	*p* < 0.05	0.438	Cuasi	0.485	Cuasi	−0.272		−0.022	

## Data Availability

The data that support the findings of this study are available on request from the corresponding author. The data are not publicly available due to privacy or ethical restrictions.
